# Placebo Effect in Levodopa‐Induced Dyskinesia: A Systematic Review and Meta‐Analysis

**DOI:** 10.1002/mdc3.70685

**Published:** 2026-05-25

**Authors:** Luiz Vinicius Silva Correa, Alexandre Martins do Nascimento, Bruno Lopes Santos‐Lobato, Henry Mauricio Chaparro‐Solano, Ignacio Fernandez Mata, Vitor Tumas

**Affiliations:** ^1^ Department of Neuroscience University of São Paulo Ribeirão Preto Brazil; ^2^ Faculdade de Medicina de Ribeirão Preto University of São Paulo Ribeirão Preto Brazil; ^3^ Department of Biochemistry Institute of Chemistry, University of São Paulo São Paulo Brazil; ^4^ Laboratório de Neuropatologia Experimental Universidade Federal do Pará Belém Brazil; ^5^ Department of Molecular Medicine Cleveland Clinic Lerner College of Medicine, Case Western Reserve University Cleveland Ohio USA; ^6^ Genome Sciences and Systems Biology Department Cleveland Clinic Research Cleveland Ohio USA

**Keywords:** levodopa‐induced dyskinesia, meta‐analysis, Parkinson's disease, placebo effect, randomized controlled trials

## Abstract

**Background:**

Levodopa‐induced dyskinesia (LID) is a complication of Parkinson's disease (PD) treatment. While the placebo effect is a known component in PD clinical trials, its specific magnitude in the context of LID is not well characterized.

**Objective:**

To quantify the magnitude of the placebo response in LID trials and to identify potential moderators of this effect.

**Methods:**

We conducted a systematic review and meta‐analysis of double‐blind, placebo‐controlled trials in patients with PD and LID. The primary outcome was the change in dyskinesia severity in the placebo arm, pooled using random‐effects model.

**Results:**

Thirty one publications, comprising 1329 patients, were included. Data were stratified by reporting methodology: 16 studies provided primary LS mean change data, 11 provided simple change and the remaining studies contributing solely to subgroup analyses. The primary meta‐analysis, revealed a statistically significant placebo response of moderate magnitude (pooled SMD = −0.48, *p* < 0.0001), with significant heterogeneity (*I* ^2^ = 47.6%). Subgroup analyses showed this corresponded to clinically measurable improvements, including a mean reduction of −6.78 points on the UDysRS and an increase of +1.27 h in “Good On Time.” Meta‐regression identified that a larger placebo effect was significantly associated with older patient age (*p* = 0.014), a higher proportion of female participants (*p* = 0.016), and shorter study duration (*p* = 0.006).

**Conclusion:**

The placebo response in LID is a consistent, clinically relevant phenomenon modulated by patient demographics and trial duration. Quantifying this response is essential for design and accurate interpretation of future trials.

Parkinson's disease (PD) is the second most common neurodegenerative disease, characterized by the onset of motor symptoms due to the loss of dopaminergic neurons in the substantia nigra in the midbrain.^1^ The gold standard treatment is levodopa, which aims to replace dopamine depleted by disease progression. Although effective, its use may lead to the onset of motor complications, such as levodopa‐induced dyskinesia (LID). It represents a key limitation of PD therapy, defined by the occurrence of involuntary choreic or dystonic movements that significantly decreases the quality of life of patients with PD,^2^, ^3^ posing a difficult clinical challenge. Its incidence is directly related to disease duration and dopaminergic therapy. Estimated LID rates are about 30% by 5 years of treatment, progressively increasing with the subsequent years.^4^, ^5^ To date, a wide range of strategies were tested to address this problem, including sparing the use of levodopa, optimization of levodopa delivery to provide more continuous dopaminergic stimulation and development of novel non‐dopaminergic adjunctive therapies to control LID.^6^

Therefore, several clinical trials have been conducted investigating therapeutic strategies to mitigate LIDs, in which placebo is a fundamental element of validation, serving as a control to differentiate the specific pharmacological effects of an intervention from the non‐specific effects of the therapeutic context. Placebo is often described as an inert substance or sham intervention with no pharmacological action. However, a more complete definition considers the placebo not as the fake treatment itself, but as the entire therapeutic ritual and the complex psychosocial context of its administration.^7^ Placebo responses are largely driven by the expectation of therapeutic benefit, a process closely linked to modulation of the dopaminergic system.^8^ For this reason, the placebo effect is of great interest in PD. Even in the setting of chronically depleted dopamine levels, the expectation of a therapeutic benefit can generate meaningful clinical improvement, highlighting its ability to engage remaining neural resources.^9^

The magnitude of the placebo response in PD is well established. In a meta‐analysis of clinical trials, the overall response rate attributable to placebo was quantified at 16% for reduction on motor symptoms.^10^ On the other hand, the placebo effect on LID is equally well known but considerably less explored in the literature, and could be related to non‐dopaminergic activation mechanisms.^11^

It would be valuable to have a broader view of the placebo effect in trials targeting the treatment of LID and to identify the main factors associated with this phenomenon. In this study, we performed a systematic review and meta‐analysis of randomized controlled trials to determine the magnitude and the main factors associated with the placebo effect on LID.

## Methods

This systematic review was performed in accordance with the Preferred Reporting Items for Systematic Reviews and Meta‐Analyses (PRISMA) guidelines.^12^ The study protocol was registered prospectively in the International Prospective Register of Systematic Reviews (PROSPERO), under registration number CRD42024569892. A systematic literature search was performed using PubMed (National Library of Sciences), Embase (Elsevier), the Cochrane Library, and Web of Science (Clarivate) databases for studies published up to May 2025. The complete search strings for each database are detailed in the Supplementary Materials.

### Eligibility Criteria and Study Selection

Studies were included if they met all the eligibility criteria: (1) the study population consisted of adult patients with PD experiencing LID; (2) the study design was a randomized, double‐blind, placebo‐controlled trial (RDBPC) published as a full‐text, peer‐reviewed article in English (review articles, case reports, and conference abstracts were excluded). No restrictions were placed on sample size or intervention duration; (3) the study reported quantitative data regarding measurement of LID severity for the placebo arm of a validated dyskinesia scale. No lower date limit was applied to the search strategy, however, this criterion naturally restricted our final inclusion to studies published from 2000 onwards.

All records identified from the database searches were imported into a reference management software for deduplication.^13^ Following this, three reviewers (LVSC, BLSL, VT) independently screened the titles and abstracts of all unique records, excluding those that did not meet the eligibility criteria. The full texts of the remaining studies were then retrieved and assessed in detail by two reviewers (LVSC, AMN). Any conflict at either screening stage was resolved through discussion to reach consensus.

### Data Extraction

Two review authors (LVSC, AMN) independently extracted data from all included studies, with any disagreements resolved by a third author (VT). The extracted information included study identification, baseline participant characteristics (number of participants in the placebo arm, mean age, sex distribution, mean disease duration); and trial details (nature of the placebo, duration of follow‐up). For the quantitative synthesis, the primary outcome was defined as the change in scores on a validated dyskinesia rating scale from baseline to the final follow‐up within the placebo group. The primary scales collected for this outcome were the Unified Dyskinesia Rating Scale (UDysRS), the Abnormal Involuntary Movement Scale (AIMS), and the Dyskinesia Rating Scale (DRS). Data for several secondary outcomes were also extracted when reported by the included studies. These secondary measures included changes from baseline in “on” time without troublesome dyskinesia, Unified Parkinson's Disease Rating Scale Part (UPDRS) part III and IV.

The risk of bias for each included study was evaluated using version 2 of the Cochrane risk‐of‐bias tool for randomized trials (RoB 2).^14^ The assessment was conducted independently by two authors (LVSC and AMN), and any disagreements were resolved through discussion with a third author (VT).

### Statistical Analysis

For the quantitative synthesis, the effect measure was the standardized mean difference (SMD) of the change from baseline in dyskinesia scores, with its corresponding standard error (SE). Utilizing the SMD allowed us to pool continuous data across trials that employed disparate clinical rating scales by standardizing their results into a uniform metric. As studies reported outcomes as either Least Squares (LS) mean change or simple mean change, we conducted separate, non‐overlapping meta‐analyses for each data type. The analysis of LS mean change was considered the primary analysis. When the SE was not reported, it was calculated from other available statistics (95% confidence intervals or p‐values) following standard formulas and methods described in the Cochrane Handbook for Systematic Reviews of Interventions.^15^ These data were pooled using a random‐effects model, with the between‐study variance (*τ* ^2^) estimated using the Restricted Maximum‐Likelihood (REML) method. Statistical heterogeneity across studies was assessed using the Cochran's *Q* test and quantified with the I^2^ statistic. To investigate sources of heterogeneity, we conducted univariable meta‐regression for pre‐specified moderators (study duration, baseline age, risk of bias, sex distribution, and baseline dyskinesia severity). A multivariable meta‐regression was also planned to assess the combined influence of significant moderators. In addition, to evaluate the influence of individual studies on the overall summary estimate, a leave‐one‐out (LOO) sensitivity analysis was performed by iteratively removing one study at a time. All meta‐analyses were performed using the metafor (version 4.8.0) package in R statistical software (version 4.5.1).

### Subgroup and Sensitivity Analyses

In addition to the primary analysis using the SMD, we conducted pre‐specified subgroup meta‐analyses to provide more clinically interpretable results in the original units of specific commonly reported scales. These analyses were also stratified by the type of reported data. For the cohort of studies providing LS mean change, we performed separate meta‐analyses for three distinct outcomes: UPDRS‐III, “Good On Time,” and the UDysRS. For studies providing simple mean change, a sufficient number of publications were available to conduct a separate analysis only for the UPDRS‐III. The statistical protocol for each of these scale‐specific subgroups was identical to our primary analysis, a random‐effects model with a REML estimator was used to pool the data, and the robustness of each result was assessed with LOO sensitivity analyses.

As an additional sensitivity analysis, we stratified studies according to their primary outcome result (positive/efficacious vs. negative/non‐efficacious) to assess whether the magnitude of the placebo response differed between trials that demonstrated active treatment efficacy and those that did not. Trials were classified as “positive” when the primary outcome showed a statistically significant difference favoring the active treatment, and “negative” otherwise. For this analysis, we performed separate random‐effects meta‐analyses for each subgroup, using identical statistical protocol as in the primary analysis. Additionally, the individual study SMDs were compared between positive and negative trials using the Mann–Whitney *U* test. Baseline characteristics (sample size, duration, age, baseline dyskinesia severity, sex distribution, and risk of bias) were also compared between subgroups using appropriate statistical tests.

## Results

### Study Selection and Characteristics

The systematic literature search initially identified 399 records. Following the screening of titles and abstracts and the subsequent full‐text review, 31^16^, ^17^, ^18^, ^19^, ^20^, ^21^, ^22^, ^23^, ^24^, ^25^, ^26^, ^27^, ^28^, ^29^, ^30^, ^31^, ^32^, ^33^, ^34^, ^35^, ^36^, ^37^, ^38^, ^39^, ^40^, ^41^, ^42^, ^43^, ^44^, ^45^, ^46^ publications met the prespecified eligibility criteria and were included in the final quantitative synthesis (Fig. 1). It is important to note that three publications reported results from separate, independent clinical trials. These were treated as distinct datasets for the purpose of analysis (Table 1). Collectively, these 34 randomized controlled trials provided data from an aggregated population of 1329 patients. Due to variations in data reporting across trials, the quantitative analysis was stratified into two main cohorts. The primary meta‐analysis incorporated 16 studies that reported LS mean change from baseline, considered the most robust estimator of effect size. A secondary meta‐analysis was conducted on 11 studies that provided simple change scores (endpoint minus baseline) or sufficient raw data to calculate them. The remaining studies, which did not report extractable change data suitable for pooling in the main analyses, contributed exclusively to specific subgroup analyses and qualitative synthesis.

**Figure 1:**
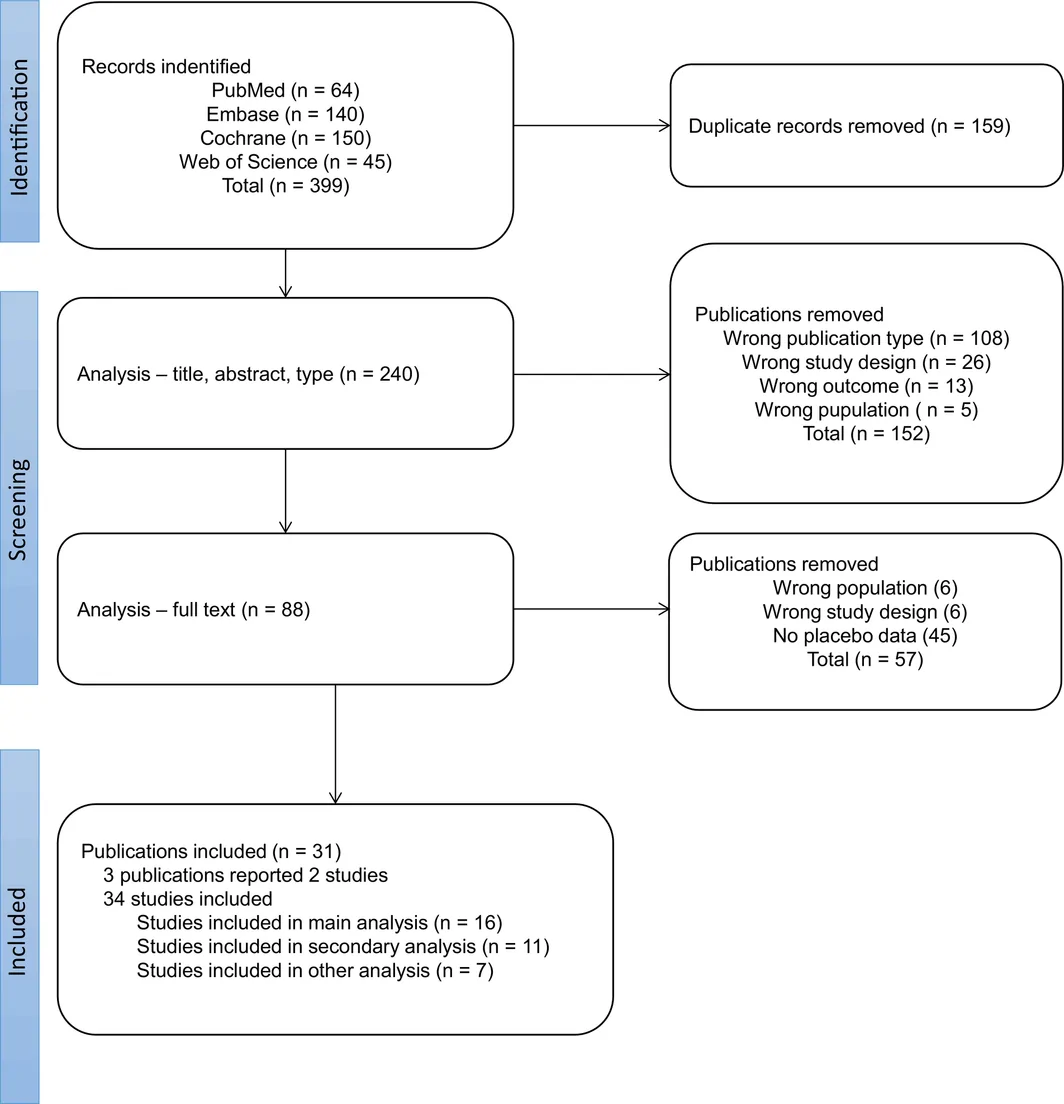
Flow diagram of study selection using preferred reporting items for systematic reviews and meta‐analyses (PRISMA) protocol.

**TABLE 1:** Summary of individual study characteristics, PD and dyskinesia duration, and extracted outcomes

Reference	Study design	Countries of recruitment	Treatment	Dose (mg)	Duration	Sample size, placebo [baseline‐final]	Age, years [mean (SD)]	Men (%)	PD duration [mean (SD)]	DYSK duration [mean (SD)]	Outcomes extracted	Outcomes reporting
Mesnage et al^30^	P/U	France	SR48692	180	9 d	12–12	65.0 (7.0)	‐	12.0 (3.0)	‐	F	Mean changes
			SR141716	20								
			SR142801	200								
Durif et al^21^	P/M	France	Clozapine	12.5–75	10 wk	24–17	63.8 (9.8)	‐	12.1 (4.4)	‐	F	Mean changes
Braz et al^19^	P/U	Brazil	Riluzole	50	1 wk	8–8	59.9 (6.6)	75.0	11.1 (4.9)	‐	F	Mean changes
Silva‐Júnior et al^38^	P/U	Brazil	Amantadine	100–200	3 wk	10–9	62.1 (9.7)	50.0	9.4 (3.0)	‐	D, F	Mean changes
Goetz et al^23^	P/M	Belgium, Bulgaria, France, Germany, Hungary, Portugal, Romania, South Africa, UK, USA	Sarizotan	2/4/10	12 wk	98–95	63.1 (8.3)	51.0	13.7 (5.8)	5.4 (3.7)	A, E, F	LS‐mean changes
Berg et al^17^ (study 1)	P/M	Germany	AFQ056	25–150	16 d	16–16	61.4 (10.3)	43.8	‐	‐	A, F	LS‐mean changes
Berg et al^17^ (study 2)	P/M	Germany	AFQ056	25–150	20 d	14–14	66.1 (6.5)	57.1	‐	‐	A, F	LS‐mean changes
Sawada et al^36^	Cr/M	Japan	Amantadine	300	27 d	32–32	63.0 (7.2)	28.6	13.4 (7.0)	‐	F	LS‐mean changes
Wolz et al^45^	P/U	Germany	Levetiracetam	2000	11 wk	14–14	67.0 (7.5)	42.9	‐	‐	A, F	Mean changes
Mizuno et al^31^	P/M	Japan	Istradefylline	20/40	12 wk	118–118	65.0 (7.6)	53.0	9.6 (5.5)	2.2 (2.1)	F	Mean changes
Goetz et al^24^	P/M	‐	Amantadine	300	8 wk	32–30	68.5 (6.9)	‐	9.4 (4.9)	3.9 (3.6)	A	Mean changes
Stocchi et al^39^	P/M	Australia, Canada, Finland, France, Germany, Italy, Japan, Spain	AFQ056	20/50/100/150/200	13 wk	64–63	64.8 (8.2)	46.9	11.9 (5.7)	4.5 (3.9)	A, F	LS‐mean changes
Borgohain et al^18^	P/M	India, Romania, Italy	Safinamide	50/100	24 wk	222–222	59.4 (9.4)	72.1	8.3 (3.8)	‐	C, E, F	LS‐mean changes
Sayin et al^37^	Cr/U	Turkey	LF‐rTMS^a^	‐	10 d	8–8	61.9 (9.0)	47.1	11.4 (4.6)	‐	B	Mean changes
Trenkwalder et al^43^ (study 1)	P/U	Canada, France, Germany, Hungary, Italy, Spain, USA	Mavoglurant	100	12 wk	25–25	66.6 (7.0)	60.0	11.8 (4.5)	4.3 (3.8)	A, F	LS‐mean changes
Trenkwalder et al^44^ (study 2)	P/U	Austria, Canada, France, Germany, Hungary, Italy, Slovakia, Spain, Switzerland, USA	Mavoglurant	150/200	12 wk	37–37	64.2 (9.0)	56.8	12.5 (5.6)	4.8 (3.3)	A, F	LS‐mean changes
Trenkwalder et al^43^	P/M	France, Germany, Italy, USA	AQW051	10/50	4 wk	23–21	63.3 (10.0)	56.5	‐	‐	A, F	LS‐mean changes
Kumar et al^27^	P/M	USA	Mavoglurant	50/100	5 wk	7–6	61.3 (8.9)	57.1	10.5 (10.5)	‐	E, F	LS‐mean changes
Tison et al^42^	P/M	Austria, France, Germany, USA	Diplagurant	50–300	4 wk	24–24	62.8 (8.3)	50.0	11.1 (3.5)	4.2 (2.3)	A, F	Mean changes
Oertel et al^32^	P/M	Germany, France, Spain, Austria, USA	Amantadine	274	13 wk	38–38	64.9 (9.1)	52.6	10.7 (4.3)	4.0 (2.6)	B, E	LS‐mean changes
Pahwa et al^33^ (EASE‐LID I)	P/M	Canada, USA	Amantadine	137/274	12 wk	58–58	65.5 (8.7)	60.3	9.0 (3.9)	3.3 (2.5)	B, E	LS‐mean changes
Elmer et al^22^ (EASE‐LID III)	P/M	Austria, France, Germany, Spain, USA	Amantadine	137/274	12 wk	38–38	64.9 (9.1)	52.6	10.7 (4.3)	4.0 (2.6)	B, E	LS‐mean changes
Svenningsson et al^40^	P/M	Sweden	IRL790	18	4 wk	4–4	65.5 (10.7)	75.0	14.2 (9.0)	‐	B, F	Mean changes
Habibi et al^25^	P/U	Iran	Vitamin D	0,025	12 wk	60–60	49.9 (11.1)	‐	7.8 (2.9)	2.4 (1.6)	F	Mean changes
Lieberman et al^28^	P/M	USA	NC001	2–24	10 wk	30–27	65.5 (7.2)	56.7	11.1 (5.6)	5.2 (3.2)	B, F	Mean changes
Corvol et al^20^	Cr/M	France	Naftazone	160	2 wk	16–16	62.3 (8.2)	75.0	10.7 (5.1)	‐	A, F	Mean changes
Meloni et al^29^	Cr/U	Italy	5‐Hydroxytryptophan	50	16 wk	12–11	65.3 (5.9)	54.5	15.3 (4.1)	‐	B, F	Mean changes
Rascol et al^34^ (ALLAY‐LID I)	P/M	Canada, France, Germany, Spain, USA	Amantadine	193/258	16 wk	28–18	66.1 (8.0)	57.1	10.6 (6.0)	4.0 (4.0)	B, E	LS‐mean changes
Rascol et al^35^ (ALLAY‐LID II)	P/M	Canada, France, Germany, Spain, USA	Amantadine	193/258	26 wk	44–25	63.5 (10.4)	59.1	9.0 (4.3)	4.5 (4.2)	B, E	LS‐mean changes
Rascol et al^34^	P/M	Austria, France, Germany, Italy, Spain, UK	Foliglurax	10/30	4 wk	52–52	67.0 (8.9)	53.8	10.0 (4.1)	‐	B, E	LS‐mean changes
Krishna et al^26^	P/U	North America, Asia, Europe	FUSA^a^	‐	12 wk	25–22	63.3 (9.2)	56.0	‐	‐	F	Mean changes
Zhang et al^46^	P/M	China	TPG	200	12 wk	50–50	68.9 (8.5)	38.0	11.6 (4.9)	‐	B, E, F	LS‐mean changes
Svenningsson et al^41^	P/M	Sweden	NLX‐112	2	8 wk	9–7	64.6 (6.3)	71.4	11.4 (4.2)	‐	B, F	LS‐mean changes
Antonini et al^16^	P/M	France, Israel, Italy, Poland, Serbia, USA	Mesdopetam	2.5/5/7.5	12 wk	39–37	64.5 (8.5)	35.9	10.3 (4.1)	‐	B	Mean changes

*Note*: A = mAIMS (modified abnormal involuntary movement scale); B = UDysRS (unified dyskinesia rating scale); C = DRS (dyskinesia rating scale); D = CDRS (clinical dyskinesia rating scale.); E = Good ON‐time; F = UPDRS III (unified parkinson's disease rating scale part III).

Abbreviations: Cr, crossover; d, days; M, multicentric; P, parallel; U, unicentric; wk, weeks.

^a^ Non‐pharmacological intervention.

Baseline demographic and clinical characteristics were extracted from all studies reporting these parameters to facilitate meta‐regression. Regarding geographic representation, the included trials were predominantly conducted in Europe and North America, with a substantially smaller number of studies recruiting from Asian, South American, and Middle Eastern populations. The overall mean age was 63.1 years (SD = 9.45), with 48.8% being male (n = 649), a mean PD duration of 9.6 years (SD = 6.0), and a mean LID duration of 2.1 years (SD = 2.0). Regarding the interventions tested against placebo, 29 of the 31 trials evaluated pharmacological agents, while two assessed non‐pharmacological treatments. The most investigated drugs were amantadine (eight studies) and mavoglurant (six studies). A comprehensive summary of individual study characteristics, PD and dyskinesia duration, and extracted outcomes is provided in Table 1. Two studies were rated to be at a high risk of bias based on the quality assessment.^27^, ^45^ A detailed assessment for all included studies can be found in the Supplementary Materials.

### Primary Meta‐Analysis of Placebo Response on Levodopa‐Induced Dyskinesia (Least Squares Mean Change)

The primary meta‐analysis, based on LS mean change data, included 16 studies. The pooled results showed a statistically significant reduction in dyskinesia in the placebo groups. The combined SMD was −0.48 (95% CI: −0.61 to −0.35; *p* < 0.0001), corresponding to a moderate effect size. Visually, the forest plot demonstrates that 11 out of 16 individual studies favored a placebo‐induced reduction in dyskinesia, with confidence intervals not crossing the line of no effect. There was evidence of moderate and statistically significant heterogeneity across the studies (*I* ^2^ = 47.6%; *τ* ^2^ = 0.02; *Q*‐test *p* = 0.004) (Fig. 2).

**Figure 2:**
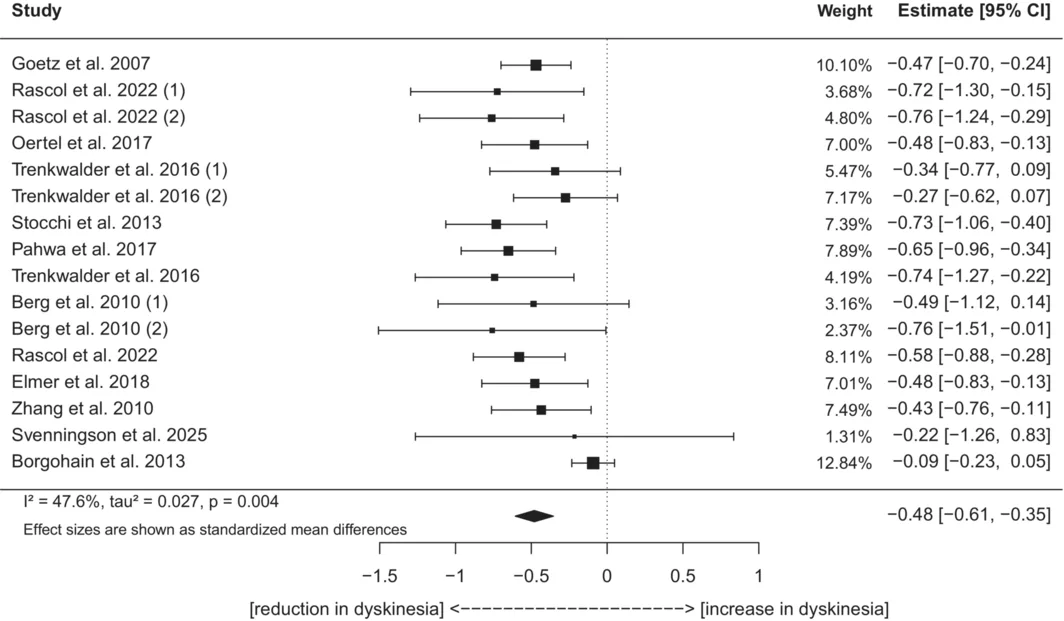
Forest plot of the random‐effects meta‐analysis of the placebo response in LID. Squares represent individual SMDs sized by study weight; horizontal lines denote 95% CIs. The diamond indicates the significant pooled moderate reduction in dyskinesia severity (−0.48). CI, confidence interval; SMD, standardized mean difference.

### Investigation of Heterogeneity and Sensitivity Analyses

The conducted univariable analyses to investigate the source of the heterogeneity between studies (*I* ^2^ = 47.6%) indicated that three moderators were significantly associated with the magnitude of the placebo effect. A smaller placebo response was observed in studies with longer durations (coefficient = 0.023, *p* = 0.002) and a higher proportion of female participants (coefficient = 1.33, *p* = 0.016). In contrast, a larger placebo response was associated with older mean patient age (coefficient = −0.05, *p* = 0.006). Moderate correlations were observed between these variables suggesting that characteristics of study designs are interrelated in the available literature. In the multivariate analysis including all three moderators simultaneously, none remained statistically significant in isolation. Although these three predictors collectively accounted for 88.6% of the total heterogeneity, reducing the residual heterogeneity to a low level (*I* ^2^ = 7.5%). Neither baseline dyskinesia severity nor risk of bias were significant moderators in the univariable analyses. The leave‐one‐out sensitivity analysis performed to identify the source of the remaining variance confirmed that the pooled SMD persisted stable and significant upon the removal of any single study. Crucially, it revealed that one study^18^ was the sole source of all between‐study heterogeneity, as its removal reduced the *I* ^2^ statistic from 47.6% to near 0% (Fig. 3).

**Figure 3:**
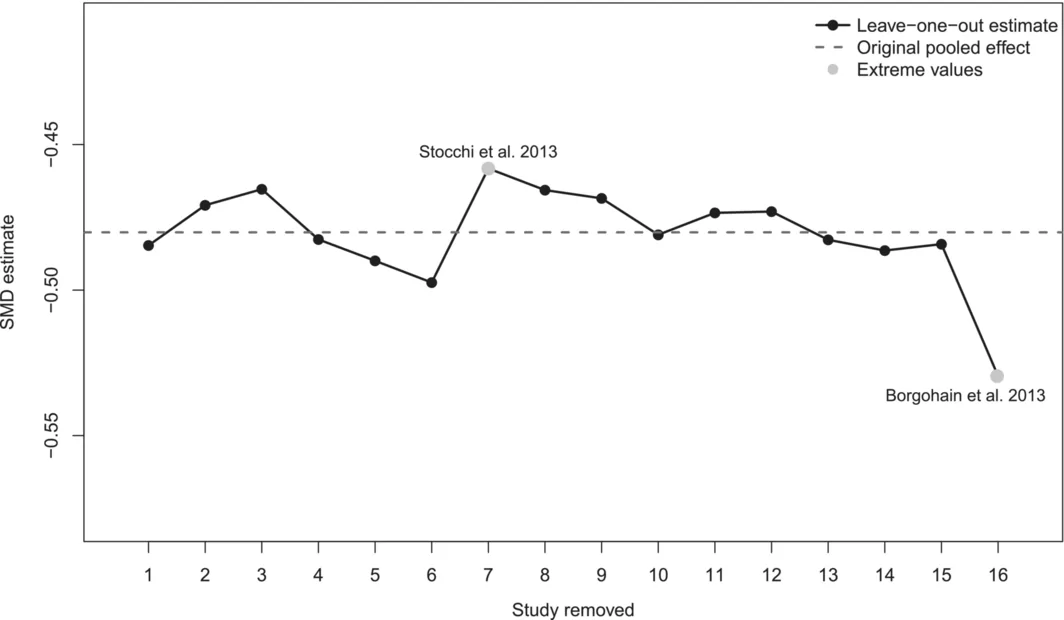
Leave‐one‐out sensitivity analysis. Each point on the plot represents the overall pooled standardized mean difference (SMD) after the removal of a single study, as indicated on the x‐axis. The horizontal red line shows the original pooled SMD from the main analysis with all 16 studies included.

### Secondary Analysis of Placebo Response on Levodopa‐Induced Dyskinesia (Simple Mean Change)

A secondary meta‐analysis was conducted on the 11 studies that provided simple mean change data. The pooled analysis also showed a statistically significant reduction in dyskinesia, with a combined SMD of −0.25 (95% CI −0.46 to −0.04; *p* = 0.012), corresponding to a small‐to‐moderate effect size. The heterogeneity across these studies was moderate (*I* ^2^ = 35.1%; *τ* ^2^ = 0.04; *Q*‐test *p* = 0.179). The subsequent meta‐regression analysis revealed that the risk of bias was a highly significant moderator of the placebo effect (*p* = 0.0009), explaining 100% of the between‐study heterogeneity (*I* ^2^ residual = 0%). Specifically, studies rated to be at a low risk of bias showed a larger and more consistent placebo effect (SMD = −0.45), whereas studies rated as “some concerns” reported smaller, non‐significant effects. Other potential moderators, including study duration, mean age, and baseline dyskinesia severity, were not significant in this analysis (Supplementary Materials).

### Placebo Response on Individual Clinical Scales

To provide more clinically interpretable results, we conducted subgroup meta‐analyses for the three most commonly reported outcome scales. For motor function, a pooled analysis of 12 studies using the UPDRS‐III showed a significant placebo effect, with a mean reduction of −1.88 points (95% CI −3.02 to −0.74; *p* = 0.0013). Substantial heterogeneity was observed (*I* ^2^ = 62%). For “Good on Time,” an analysis of 10 studies revealed a statistically significant mean increase of +1.27 h (95% CI 0.90 to 1.65; *p* < 0.0001) in the placebo group, with moderate heterogeneity (*I* ^2^ = 51%). Finally, for the outcome of dyskinesia measured specifically by the UDysRS, an analysis of eight studies found a highly significant placebo‐induced reduction of −6.78 points (95% CI −8.34 to −5.23; *p* < 0.0001), with low heterogeneity (*I* ^2^ = 24%) (Fig. 4).

**Figure 4:**
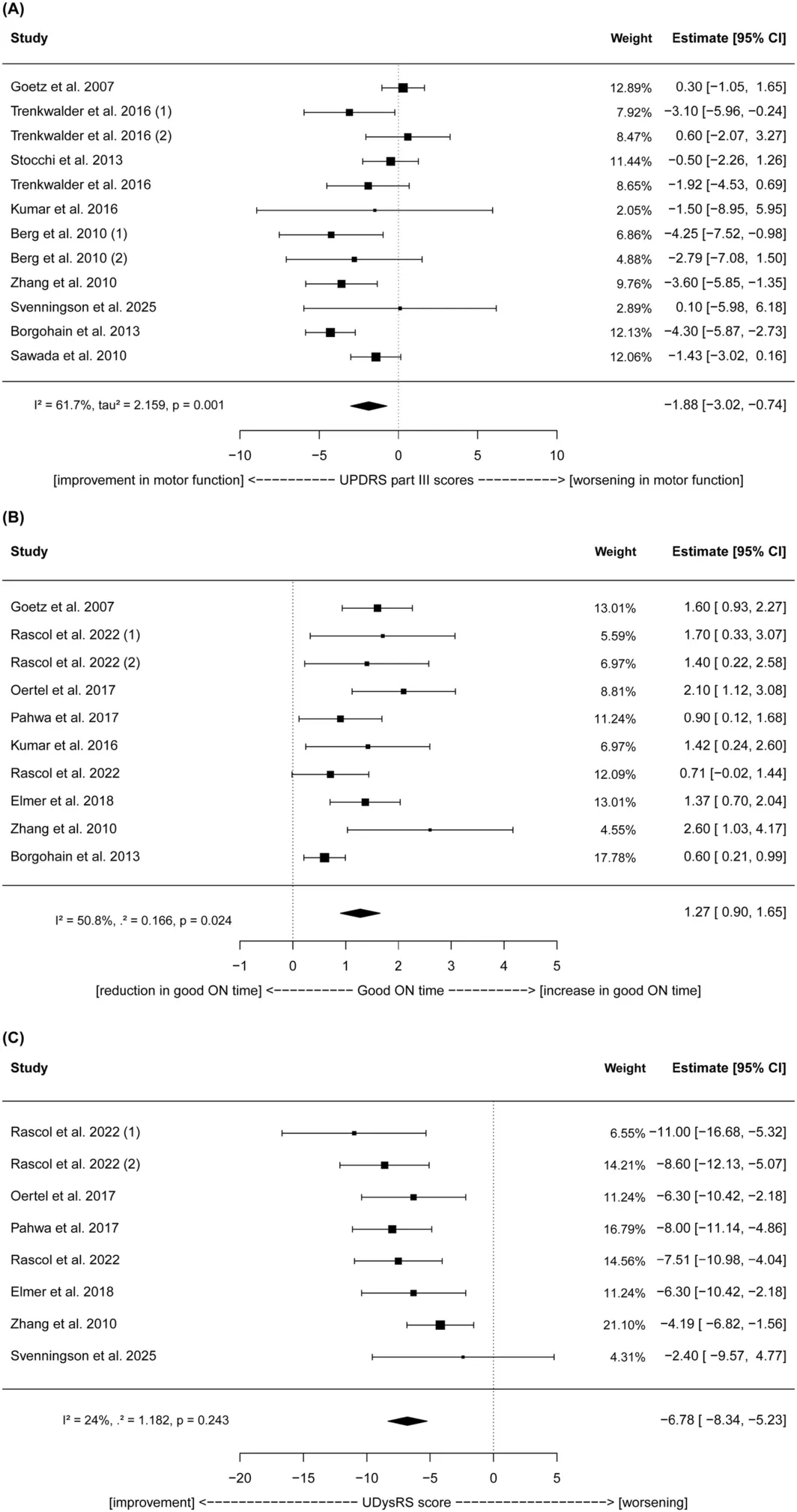
Forest plots of the placebo response on individual clinical scales. Results of three separate random‐effects meta‐analyses for the most commonly reported outcomes. (A) Placebo effect on motor function, measured by the change in the Unified Parkinson's disease rating scale (UPDRS‐III) score, based on data from 12 studies. The pooled estimate showed a significant mean reduction of −1.88 points (95% CI −3.02 to −0.74). (B) Placebo effect on functional time, measured by the change in “Good On Time,” based on data from 10 studies. The pooled estimate showed a significant mean increase of +1.27 (95% CI 0.90 to 1.65). (C) Placebo effect on dyskinesia, measured by the change in the Unified Dyskinesia Rating Scale (UDysRS) score, based on data from eight studies. The pooled estimate showed a significant mean reduction of −6.78 points (95% CI −8.34 to −5.23).

### Sensitivity Analysis: Comparison of Placebo Response between Trials with and without Demonstrated Treatment Efficacy

The placebo response did not differ between positive and negative trials. The pooled SMD was −0.484 (95% CI −0.664 to −0.305) for positive trials (*k* = 10) and − 0.482 (95% CI −0.623 to −0.341) for negative trials (*k* = 6). The Mann–Whitney *U* test comparing individual study SMDs between the two groups confirmed no significant difference (*p* = 0.91).

Regarding baseline characteristics, negative trials had significantly lower baseline dyskinesia severity (mean 2.43 for negative vs. 3.26 for positive; *p* = 0.02). No other significant differences were observed for sample size (*p* = 0.91), study duration (*p* = 0.40), age (*p* = 0.55), or risk of bias (*p* = 0.30).

## Discussion

### The Placebo Effect as an Integral Component of LID Outcomes

A statistically significant placebo response of moderate magnitude was consistently identified in patients with LID across the various analyses in this systematic review. Our primary analysis of 16 studies using LS mean change data quantified this as a moderate effect, with a pooled SMD of −0.48 (95% CI −0.61 to −0.35; *p* < 0.0001). Despite significant heterogeneity (*I* ^2^ = 47.6%), the pooled estimate remained robust across our sensitivity analyses. Notably, the LOO analysis identified the study by Borgohain et al^18^ as the primary driver of this variance. This trial differed from the typical profile of included studies due to its extended duration (24 weeks), large sample size (>200 participants), and a demographic skew towards a predominantly male population (75%). Also, this was the only trial that used DRS (dyskinesia rating scale) as a scale to measure dyskinesias. The exclusion of this single outlier resulted in a substantial reduction of heterogeneity, reinforcing the consistency of the placebo signal across the remaining literature.

In clinically interpretable units, these pooled estimates translate to significant improvements, including a mean reduction of −6.78 points on the UDysRS and an increase of +1.27 h in “Good on Time.” Relative to the weighted baseline severity of the included trials, these changes correspond to a substantial 45.1% reduction in dyskinesia severity and a 21.3% gain in functional “ON” time. The specificity of this response is highlighted by the comparatively smaller 10.8% improvement in motor parkinsonism (UPDRS‐III). This divergence in magnitude offers implications for trial design regarding the susceptibility of different instruments to the placebo effect. While the UDysRS demonstrated a remarkably high placebo response approaching 50%, the diary‐based “Good on Time” measure showed a more conservative response profile. This suggests that despite the perceived subjectivity of patient diaries, functional time metrics may be less prone to placebo‐induced inflation than composite rating scales in these specific populations. Consequently, identifying outcome measures with lower placebo sensitivity, such as functional “ON” time, serves as a crucial strategy for improving future anti‐dyskinetic trials.

The clinical impact of the robust placebo response that we quantified must be interpreted in light of additional findings from this review. Specifically, the interpretation of the placebo effect requires acknowledging its relative and context‐dependent nature.^8^, ^9^ The magnitude of this response may vary according to the specific comparator treatments utilized, driven by differences in the anticipated efficacy and the expectations associated with the active intervention being tested. In some trials, the improvement observed in the placebo group was numerically superior to that observed in the active treatment arm,^21^, ^39^, ^44^ illustrating the unpredictable variability of placebo responses in specific study contexts. However, when we stratified trials according to their primary outcome, the pooled placebo response was virtually identical between studies that demonstrated active treatment efficacy and those that did not (SMD = −0.48 for both groups; *p* = 0.91). This indicates that a high placebo response alone does not explain the failure to detect treatment effects in negative trials. Instead, exploratory analyses revealed that negative trials enrolled patients with significantly lower baseline dyskinesia severity, which may have limited the available margin for improvement. Taken together, these findings suggest that while the placebo response is consistently large and represents a fundamental challenge in drug development for LID, its contribution to trial failure is likely modulated by other factors.

### Contextualizing the Predictors of the Placebo Effect

Previous literature on the placebo effect in PD clinical trials has identified several predictors of a larger response, including higher baseline motor severity, longer disease duration, and the presence of motor fluctuations.^47^, ^48^ Although most of these observations derive from studies conducted in PD patients without a specific focus on dyskinesia, these predictors remain relevant for understanding the variability in placebo responses observed in our analysis.

Our findings on the moderators of the placebo response partially aligns with the seminal analysis by Goetz et al,^10^ particularly regarding demographic factors. A key point of convergence is the influence of age: our meta‐regression identified older patient age as a significant predictor of a larger placebo effect, a finding that aligns with their report. One possible explanation is that older individuals may rely more on learned associations and conditioned expectations from prolonged treatment histories, which could amplify expectancy‐driven responses. Although direct evidence in dyskinesia trials is lacking, aging research in reward learning shows that older adults exhibit alterations in dopaminergic regulation and anticipatory value signals in prefrontal–limbic circuits.^49^

Regarding baseline dyskinesia severity, the comparison is more nuanced. Consistent with our results, Goetz et al reported that initial symptom severity did not influence the overall *likelihood* of a placebo response. However, their analysis of individual patient data revealed a specific pattern where patients with lower baseline scores were more prone to worsening, suggesting a potential floor effect. In contrast, our meta‐regression found no significant association between baseline dyskinesia severity and the magnitude of the placebo effect across studies. This discrepancy highlights a fundamental methodological distinction: while Goetz et al's use of individual data allowed for the detection of granular patterns in mild cases, our aggregated, study‐level analysis suggests that, for the average clinical cohort, the objective intensity of motor symptoms is not a primary driver of the placebo response. Instead, the phenomenon appears more closely linked to the aforementioned demographic and potentially psychological factors.

Our analyses further suggest that both study duration and the sex composition of the sample act as key moderators of the placebo response. We observed a moderate correlation between these variables (*r* = 0.35), indicating a systematic tendency for longer‐duration studies to recruit higher proportions of male participants. While no individual variable retained independent statistical significance in the multivariate model, likely due to this collinearity, the combined set of predictors explained a substantial 88.6% of the between‐study heterogeneity. This finding implies that these factors do not operate in isolation but rather reflect intrinsic patterns in trial design within the LID literature. From a practical perspective, these results suggest that the magnitude of the placebo response is most pronounced in shorter trials and in cohorts with a higher proportion of female participants. Consequently, the inter‐relation between study duration and sex composition represents a critical source of variance that should be accounted for when interpreting efficacy thresholds in future anti‐dyskinetic trials.

### Evaluating Open‐Label Results against the Placebo Benchmark

Our quantitative analysis of the placebo response provides a crucial benchmark for interpreting the findings of uncontrolled, single‐arm studies, which are common in the preliminary assessment of novel therapies. These open‐label designs, by nature, cannot differentiate the specific pharmacological effect from the contextual and non‐pharmacological effects that our meta‐analysis has quantified. An example is the open‐label study previously published by our group,^50^ which investigated doxycycline for LID in eight patients. The study reported a significant mean reduction of 11 points on the UDysRS total score after 12 weeks. These results must be interpreted with caution. Our subgroup analysis identified an average placebo‐induced reduction of −6.78 points on the UDysRS. As we acknowledged in the original publication, the observed benefit is very close to the placebo effect reported in other large trials.

Similarly, the study by Baizabal‐Carvallo et al^51^ on intestinal decontamination in 14 patients reported a significant improvement in dyskinesia, with the UPDRS IV total score decreasing 6.28 points. Although the scales differ, the degree of improvement again approximates the placebo effect quantified in our meta‐analysis. While these novel interventions warrant further investigation, these comparisons strongly suggest that a substantial portion of the therapeutic benefit reported in such uncontrolled studies is attributable to the robust contextual effects identified in our meta‐analysis.

### Strengths and Limitations

The primary strength of this study lies in the systematic quantification of the placebo response in LID. To our knowledge, this is the first meta‐analysis to provide a robust, pooled estimate of this effect, addressing a gap in the literature where this magnitude had remained unquantified. The methodological rigor is evidenced by our comprehensive sensitivity analyses, which successfully identified the source of statistical heterogeneity and confirmed the stability of the placebo signal across the remaining literature.

However, several limitations must be acknowledged. First, a primary conceptual limitation is that the pooled estimate reflects the total change observed in the placebo arm, not a pure psychobiological placebo effect. This metric is inherently a composite, amalgamating the expectation response with non‐specific factors such as the natural fluctuation of dyskinesias and statistical regression to the mean. Second, our reliance on aggregated study‐level data, rather than individual patient data, restricts our ability to explore granular patient‐level predictors. Unlike Goetz et al,^10^ who identified lower daily levodopa doses as a predictor of greater placebo response, our study‐level design precluded the evaluation of complex covariates such as levodopa daily dose or cumulative drug exposure. This methodological constraint likely also explains why our moderator analysis could not replicate the specific association between lower baseline severity and reduced placebo response reported in their cohort. Third, combining varied clinical scales (eg, UDysRS, AIMS) introduces clinical heterogeneity. While using the Standardized Mean Difference (SMD) allowed us to pool these data, the SMD is highly sensitive to sample variance; consequently, trials with narrower standard deviations may artificially inflate the pooled effect size, complicating the interpretation of absolute clinical magnitude. Finally, the generalizability of our findings is constrained by the geographic distribution of the included trials, which were predominantly conducted in North American and European centers. Consequently, data regarding the placebo response in Asian, South American, and African populations remains sparse, limiting the extrapolation of these results to a globally diverse patient cohort.

## Conclusion

In conclusion, this meta‐analysis quantitatively establishes the placebo response as a moderate and integral component of outcomes in LID clinical trials. This finding compels a shift in perspective, underscoring the importance of researchers treating the placebo effect not as a mere statistical nuisance but as a substantial therapeutic phenomenon in its own right. Future investigations should therefore move beyond simply controlling for this effect and begin to actively identify its predictors, being patient‐related factors or trial design elements, to enhance the efficiency of future trials and the interpretation of true treatment efficacy.

## Author Roles

(1) Research Project: A. Conception; B. Organization; C. Execution; (2) Statistical Analysis: A. Design; B. Execution; C. Review and Critique; (3) Manuscript Preparation: A. Writing of the first draft; B. Review and Critique.

L.V.S.C.: 1B, 1C, 2A, 2B, 2C, 3A, 3B.

A.M.N.: 1B, 1C, 2C, 3A, 3C.

B.L.S.L.: 1C, 2C, 3B.

H.M.C.S.: 2C, 3B.

I.F.M.: 2C, 3B.

V.T.: 1A, 1B, 1C, 2C, 3B.

## Disclosures

**Ethical Compliance Statement:** We confirm that we have read the Journal's position on issues involved in ethical publication and affirm that this work is consistent with those guidelines. As this study is a meta‐analysis of publicly available aggregated data, neither Institutional Review Board approval nor informed patient consent was required.

## Financial Disclosures and Conflicts of Interest

Author disclosures are available in the Supporting Information.

## Supporting information


**TABLE S2.** Risk of bias assessment of the included studies according to the Cochrane RoB 2 tool. The risk of bias was evaluated across five domains: (D1) bias arising from the randomization process; (D2) bias due to deviations from intended interventions; (D3) bias due to missing outcome data; (D4) bias in measurement of the outcome; and (D5) bias in selection of the reported result. Colors represent the risk level: green indicates low risk of bias, yellow indicates some concerns, and red indicates high risk of bias. The overall risk rating follows the Cochrane methodology, where the highest risk in any single domain determines the overall study classification.
**Figure S5.** Funnel plot‐LS mean change. The vertical dashed line represents the pooled effect size, and the diagonal lines indicate the 95% confidence intervals. Each blue circle represents an individual study plotted by its effect size (SMD) and standard error to assess publication bias. Results of Egger's test (*t* = −3.729, *p* = 0.0022) are shown. LS, least squares; SMD, standardized mean difference.
**Figure S6.** Forest plot of simple mean changes meta‐analysis. Squares represent individual SMDs sized by study weight; horizontal lines denote 95% CIs. The diamond indicates the significant pooled reduction in dyskinesia (−0.25 [−0.46, −0.04]). CI, confidence interval; SMD, standardized mean difference.
**Figure S7.** Simple mean change leave‐one‐out sensitivity analysis. The plot shows the stability of the original pooled effect (red dashed line) when each individual study is removed. Black points represent the new SMD estimate for each iteration, with gray points highlighting extreme values (eg, Durif et al 2004 and Antonini et al 2025). SMD, standardized mean difference.
**Figure S8.** Funnel plot of the simple mean change analysis. The vertical dashed line represents the pooled effect size, with diagonal lines indicating the 95% confidence intervals. Individual studies are plotted by effect size (SMD) and standard error to evaluate publication bias. SE, standard error; SMD, standardized mean difference.
**Figure S9.** Forest plot of the meta‐analysis for UPDRS part III scores. Squares represent the effect sizes of individual studies, and horizontal lines denote 95% CIs. The diamond indicates the pooled estimate (−2.24). Negative values indicate improvement in motor function. CI, confidence interval; UPDRS, Unified Parkinson's disease rating scale.

**Data S1** Supporting Information.

## Data Availability

The data analyzed in this study were extracted from previously published clinical trials and are subject to the original publications’ copyright. Therefore, the datasets are not publicly shared but are available from the corresponding author upon request. All statistical analyses were performed in R, and the code used to generate the results is publicly available at GitHub on (https://github.com/Vinicius-Crr/placebo_effect_lid_meta_analysis) repository.
