# Supplementary material for: Placebo Effect in Levodopa‐Induced Dyskinesia: A Systematic Review and Meta‐Analysis
Source: Mov Disord Clin Pract. 2026 May 25;13(10):2365–78. doi: 10.1002/mdc3.70685 (PMC13339215; doi:10.1002/mdc3.70685)

Supplementary Materials

1. Search strings:

PUBMED: (((Parkinson* disease) AND (levodopa-induced dyskinesia)) AND (treatment OR intervention)) AND (placebo)

EMBASE: ('parkinsons disease'/exp OR 'parkinsons disease') AND ('levodopa-induced dyskinesia'/exp OR 'levodopa-induced dyskinesia') AND ('intervention'/exp OR 'intervention' OR 'therapy'/exp OR 'therapy' OR 'treatment'/exp OR 'treatment') AND ('placebo'/exp OR 'placebo')

COCHRANE: (Parkinson*) AND ("levodopa-induced dyskinesia"):ti,ab,kw AND (treatment OR intervention OR therapy):ti,ab,kw AND (placebo):ti,ab,kw

Web of science: Parkinson's disease (All Fields) and levodopa-induced dyskinesia (All Fields) and placebo (All Fields) and treatment OR therapy OR intervention (All Fields)

Table 2: ROB2 assessment:

REF_ID	D1	D2	D3	D4	D5	OVERALL
MESNAGE 2004	Green	Green	Green	Green	Green	Green
DURIF 2004	Yellow	Green	Green	Green	Green	Yellow
BRAZ 2004	Green	Green	Yellow	Green	Green	Yellow
SILVA-JUNIOR 2005	Yellow	Green	Green	Green	Green	Yellow
GOETZ 2007	Green	Green	Green	Green	Green	Green
BERG 2010	Green	Green	Green	Yellow	Green	Yellow
SAWADA 2010	Green	Green	Green	Green	Yellow	Yellow
WOLZ 2010	Green	Green	Red	Yellow	Green	Red
MIZUNO 2010	Yellow	Yellow	Green	Green	Green	Yellow
GOETZ 2012	Green	Green	Green	Green	Green	Green
STOCCHI 2013	Green	Green	Green	Green	Green	Green
BORGOHAIN 2013	Green	Green	Green	Green	Green	Green
SAYIN 2014	Yellow	Green	Green	Green	Green	Yellow
TRENKWALDER 2016	Yellow	Green	Green	Green	Green	Yellow
TRENKWALDER 2016	Yellow	Green	Green	Green	Green	Yellow
KUMAR 2016	Yellow	Yellow	Green	Green	Green	Red
TISON 2016	Green	Green	Green	Green	Green	Green
OERTEL 2017	Green	Green	Yellow	Green	Green	Yellow
PAHWA 2017	Green	Green	Green	Green	Green	Green
ELMER_2018	Green	Green	Green	Green	Green	Green
SVENNINGSON 2018	Green	Green	Green	Green	Green	Green
HABIBI 2018	Yellow	Green	Green	Green	Green	Yellow
LIEBERMAN 2019	Green	Green	Green	Green	Green	Green
CORVOL 2019	Yellow	Green	Green	Green	Green	Green
MELONI 2020	Green	Yellow	Green	Green	Green	Yellow
RASCOL 2022	Green	Green	Green	Green	Green	Green
RASCOL 2022_1	Yellow	Green	Green	Green	Green	Yellow
KRISHNA 2023	Green	Yellow	Green	Green	Green	Yellow
ZHANG 2024	Green	Green	Green	Green	Green	Green
SVENNINGSON 2025	Green	Green	Green	Green	Green	Green
ANTONINI 2025	Green	Green	Green	Green	Green	Green

Figure 5: Funnel plot - LS mean change

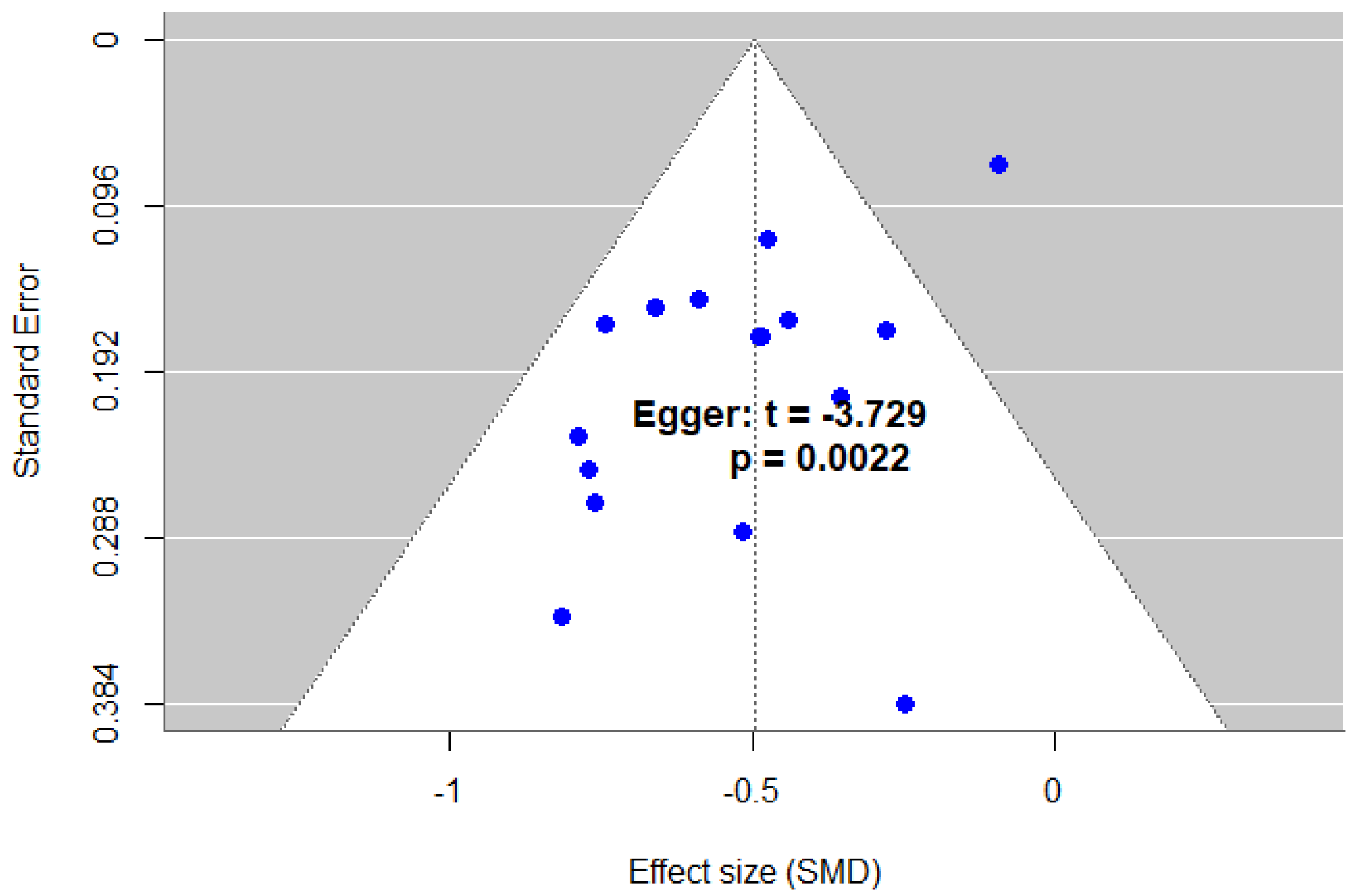

Figure 6: Simple mean change meta analysis:

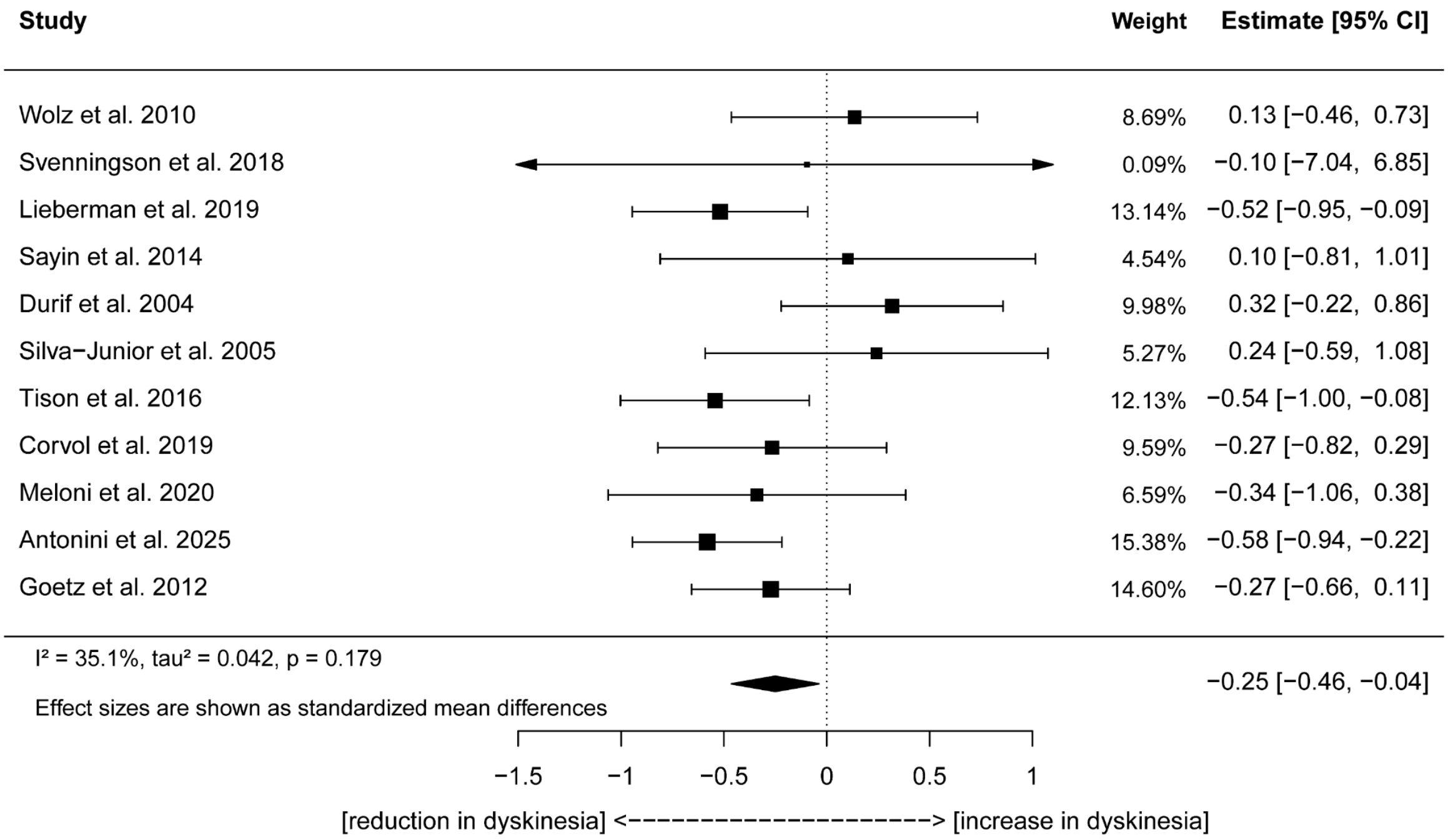

Figure 7: Simple mean change leave-one-out analysis:

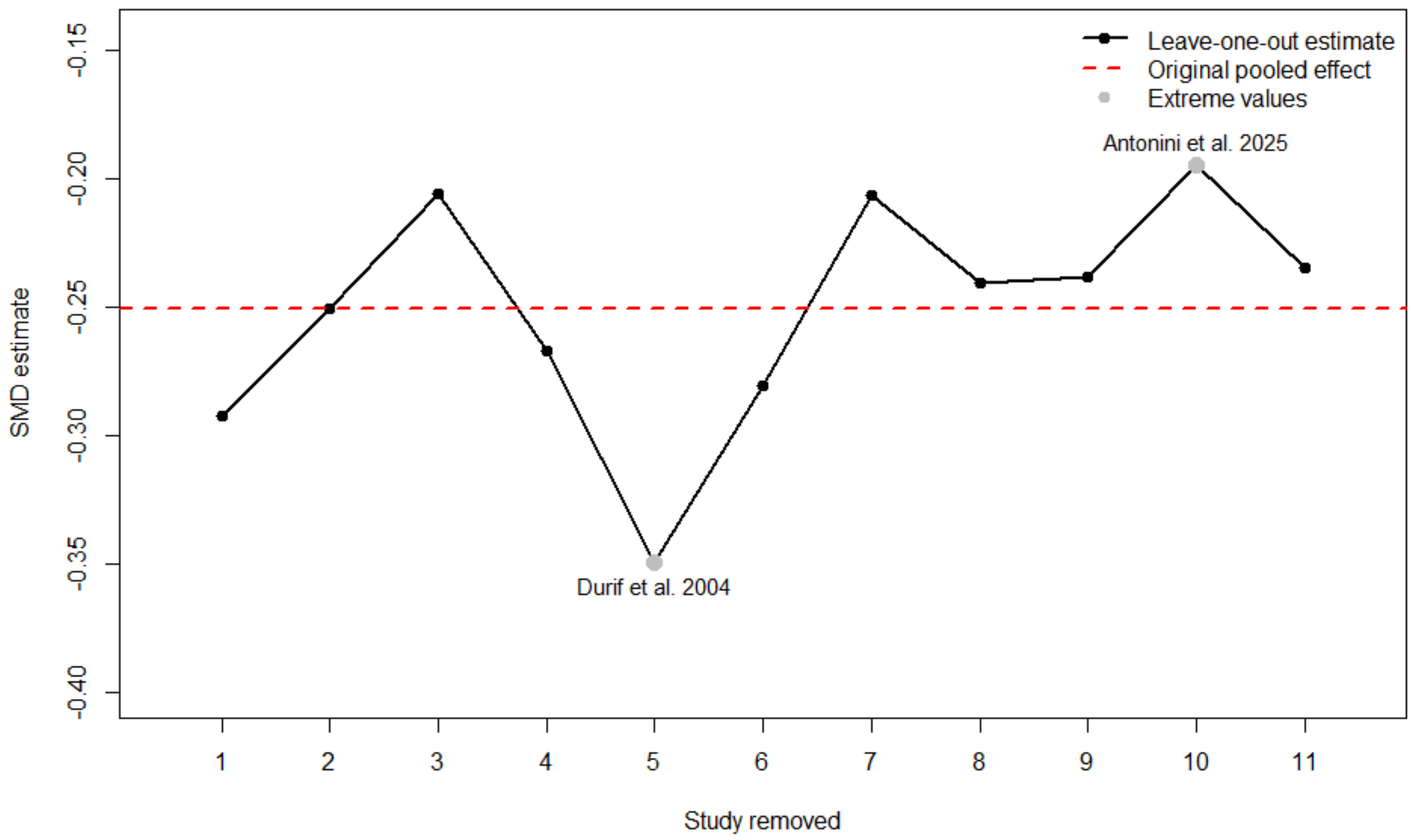

Figure 8: Funnel plot - Simple mean change

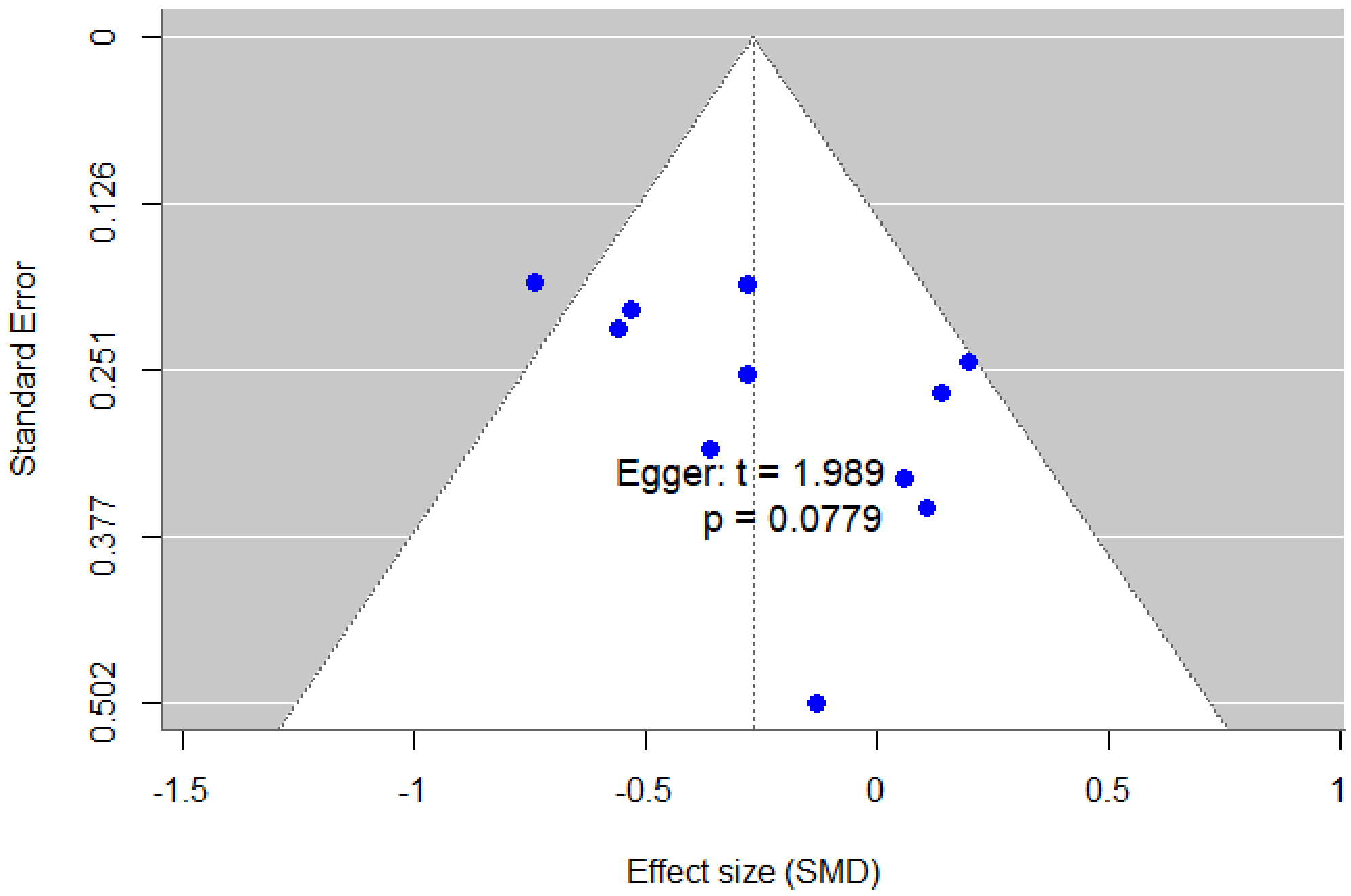

Figure 9: Simple mean change UPDRS-III meta-analysis

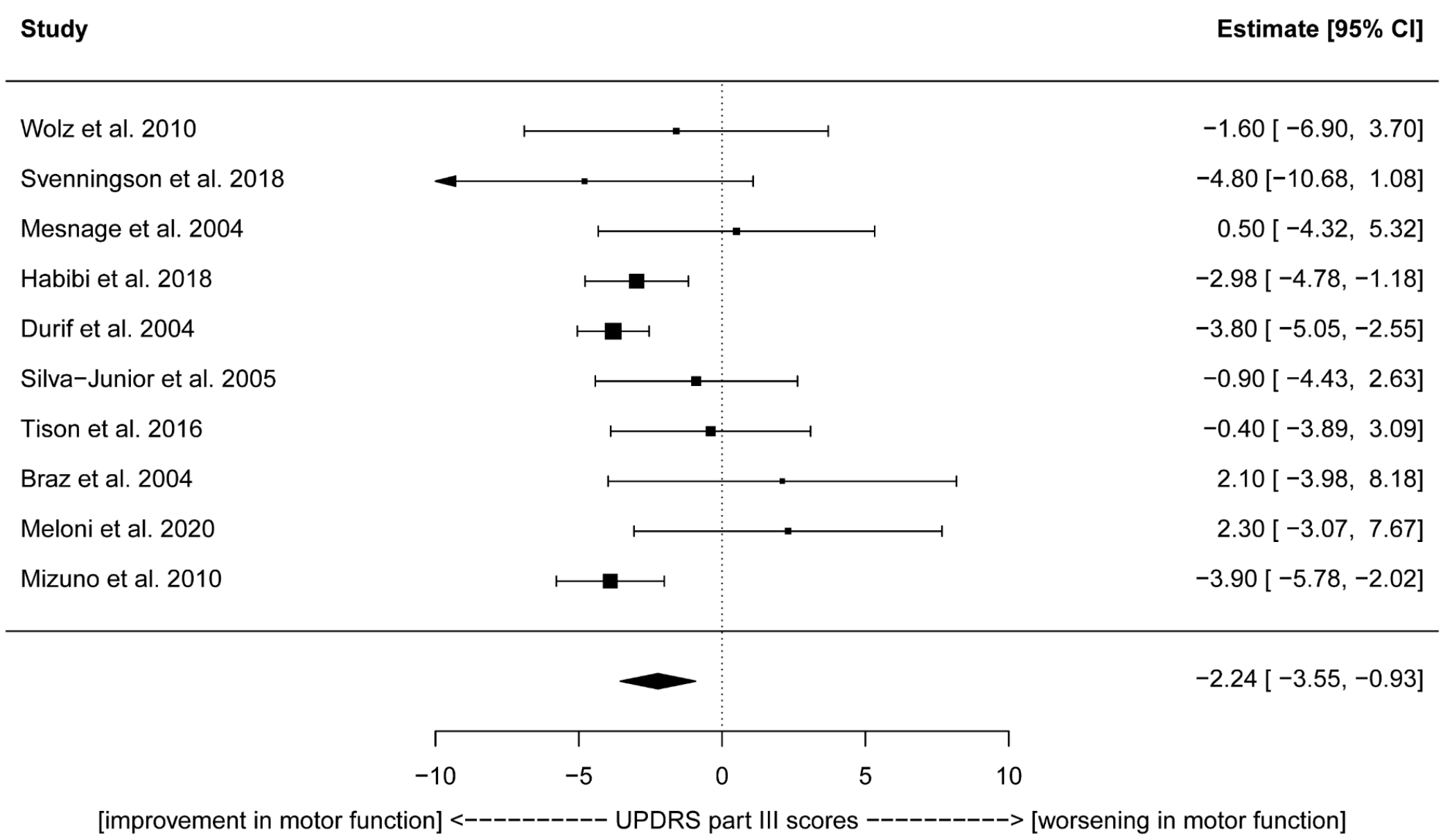

Supplement: Supplementary file 1 [file MDC3-13-2365-s001.pdf]
