# Supplementary material for: Placebo Effect in Levodopa‐Induced Dyskinesia: A Systematic Review and Meta‐Analysis
Source: Mov Disord Clin Pract. 2026 May 25:10.1002/mdc3.70685. Online ahead of print. doi: 10.1002/mdc3.70685 (PMC13339215; doi:10.1002/mdc3.70685)

Supplementary Materials

1. Search strings:

PUBMED: (((Parkinson* disease) AND (levodopa-induced dyskinesia)) AND (treatment OR intervention)) AND (placebo)

EMBASE: ('parkinsons disease'/exp OR 'parkinsons disease') AND ('levodopa-induced dyskinesia'/exp OR 'levodopa-induced dyskinesia') AND ('intervention'/exp OR 'intervention' OR 'therapy'/exp OR 'therapy' OR 'treatment'/exp OR 'treatment') AND ('placebo'/exp OR 'placebo')

COCHRANE: (Parkinson*) AND ("levodopa-induced dyskinesia"):ti,ab,kw AND (treatment OR intervention OR therapy):ti,ab,kw AND (placebo):ti,ab,kw

Web of science: Parkinson's disease (All Fields) and levodopa-induced dyskinesia (All Fields) and placebo (All Fields) and treatment OR therapy OR intervention (All Fields)

Table 2: ROB2 assessment:

REF_ID	D1	D2	D3	D4	D5	OVERALL
MESNAGE 2004	Green	Green	Green	Green	Green	Green
DURIF 2004	Yellow	Green	Green	Green	Green	Yellow
BRAZ 2004	Green	Green	Yellow	Green	Green	Yellow
SILVA-JUNIOR 2005	Yellow	Green	Green	Green	Green	Yellow
GOETZ 2007	Green	Green	Green	Green	Green	Green
BERG 2010	Green	Green	Green	Yellow	Green	Yellow
SAWADA 2010	Green	Green	Green	Green	Yellow	Yellow
WOLZ 2010	Green	Green	Red	Yellow	Green	Red
MIZUNO 2010	Yellow	Yellow	Green	Green	Green	Yellow
GOETZ 2012	Green	Green	Green	Green	Green	Green
STOCCHI 2013	Green	Green	Green	Green	Green	Green
BORGOHAIN 2013	Green	Green	Green	Green	Green	Green
SAYIN 2014	Yellow	Green	Green	Green	Green	Yellow
TRENKWALDER 2016	Yellow	Green	Green	Green	Green	Yellow
TRENKWALDER 2016	Yellow	Green	Green	Green	Green	Yellow
KUMAR 2016	Yellow	Yellow	Green	Green	Green	Red
TISON 2016	Green	Green	Green	Green	Green	Green
OERTEL 2017	Green	Green	Yellow	Green	Green	Yellow
PAHWA 2017	Green	Green	Green	Green	Green	Green
ELMER_2018	Green	Green	Green	Green	Green	Green
SVENNINGSON 2018	Green	Green	Green	Green	Green	Green
HABIBI 2018	Yellow	Green	Green	Green	Green	Yellow
LIEBERMAN 2019	Green	Green	Green	Green	Green	Green
CORVOL 2019	Yellow	Green	Green	Green	Green	Green
MELONI 2020	Green	Yellow	Green	Green	Green	Yellow
RASCOL 2022	Green	Green	Green	Green	Green	Green
RASCOL 2022_1	Yellow	Green	Green	Green	Green	Yellow
KRISHNA 2023	Green	Yellow	Green	Green	Green	Yellow
ZHANG 2024	Green	Green	Green	Green	Green	Green
SVENNINGSON 2025	Green	Green	Green	Green	Green	Green
ANTONINI 2025	Green	Green	Green	Green	Green	Green

Figure 5: Funnel plot - LS mean change

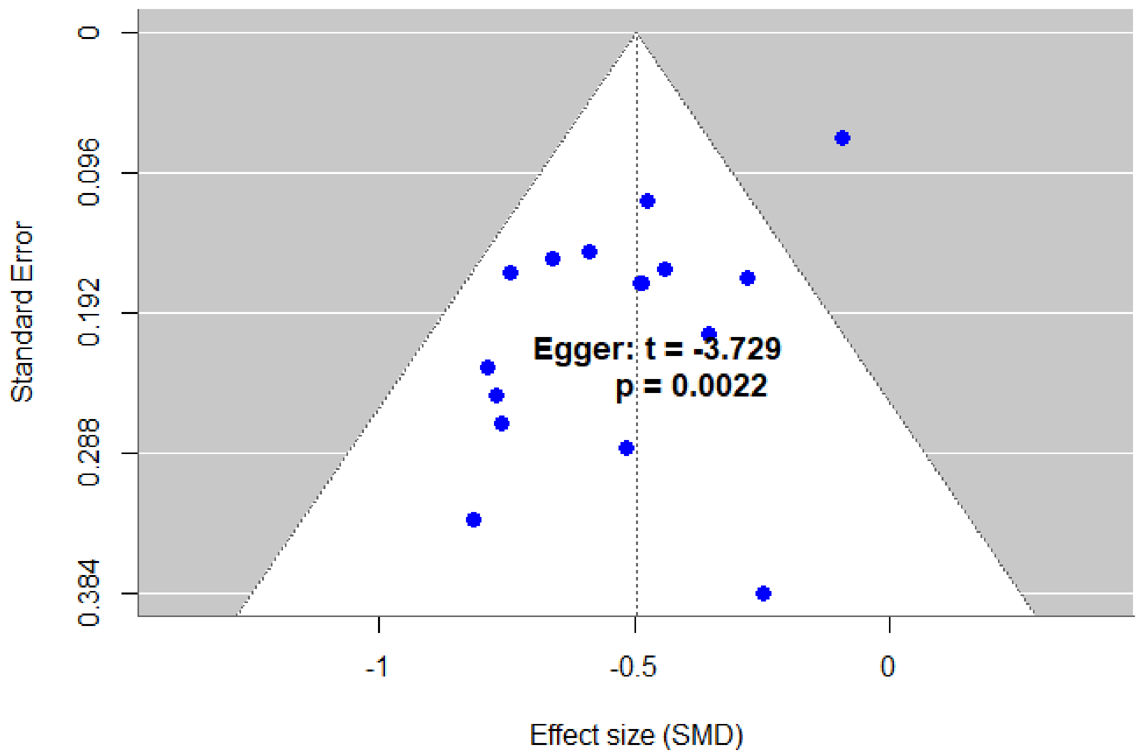

Figure 6: Simple mean change meta analysis:

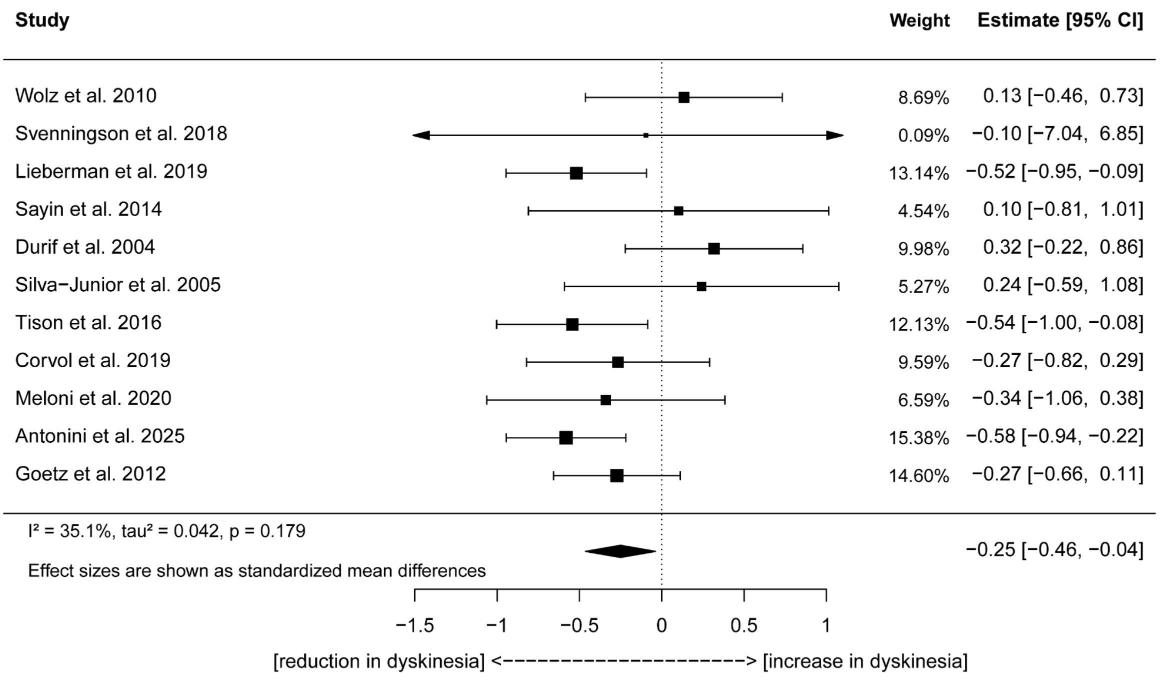

Figure 7: Simple mean change leave-one-out analysis:

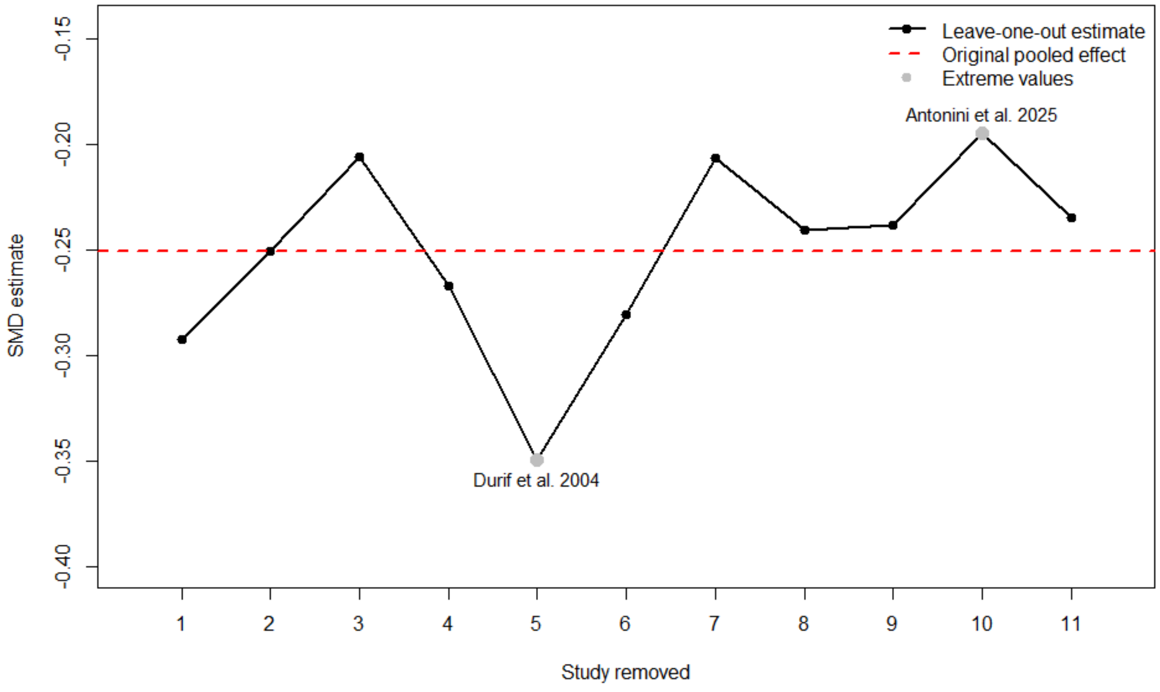

Figure 8: Funnel plot - Simple mean change

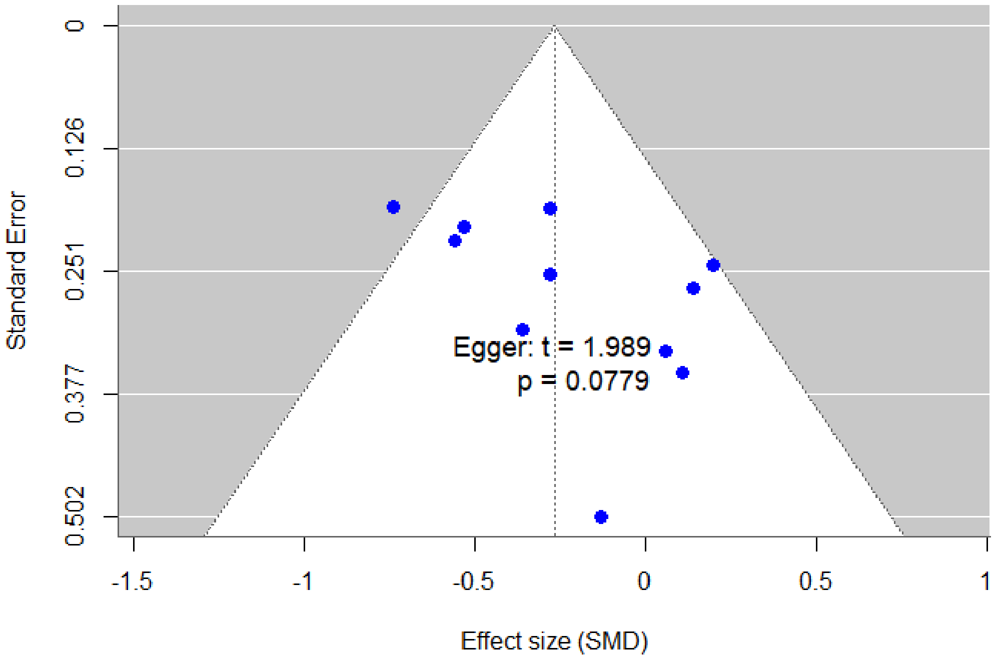

Figure 9: Simple mean change UPDRS-III meta-analysis

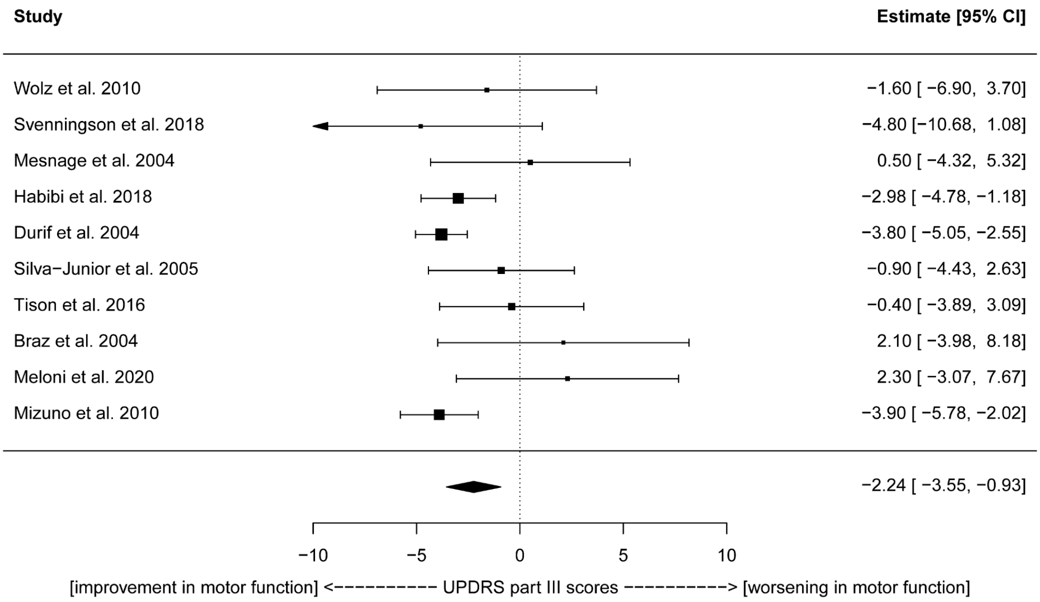

Supplement: Supplementary file 1 — TABLE S2. Risk of bias assessment of the included studies according to the Cochrane RoB 2 tool. The risk of bias was evaluated across five domains: (D1) bias arising from the randomization process; (D2) bias due to deviations from intended interventions; (D3) bias due to missing outcome data; (D4) bias in measurement of the outcome; and (D5) bias in selection of the reported result. Colors represent the risk level: green indicates low risk of bias, yellow indicates some concerns, and red indicates high risk of bias. The overall risk rating follows the Cochrane methodology, where the highest risk in any single domain determines the overall study classification. Figure S5. Funnel plot‐LS mean change. The vertical dashed line represents the pooled effect size, and the diagonal lines indicate the 95% confidence intervals. Each blue circle represents an individual study plotted by its effect size (SMD) and standard error to assess publication bias. Results of Egger's test (t = −3.729, p = 0.0022) are shown. LS, least squares; SMD, standardized mean difference. Figure S6. Forest plot of simple mean changes meta‐analysis. Squares represent individual SMDs sized by study weight; horizontal lines denote 95% CIs. The diamond indicates the significant pooled reduction in dyskinesia (−0.25 [−0.46, −0.04]). CI, confidence interval; SMD, standardized mean difference. Figure S7. Simple mean change leave‐one‐out sensitivity analysis. The plot shows the stability of the original pooled effect (red dashed line) when each individual study is removed. Black points represent the new SMD estimate for each iteration, with gray points highlighting extreme values (eg, Durif et al 2004 and Antonini et al 2025). SMD, standardized mean difference. Figure S8. Funnel plot of the simple mean change analysis. The vertical dashed line represents the pooled effect size, with diagonal lines indicating the 95% confidence intervals. Individual studies are plotted by effect size (SMD) and standard error [file MDC3-9999-0-s001.pdf]
